# Contrast-Enhanced Mammography versus Breast Magnetic Resonance Imaging: A Systematic Review and Meta-Analysis

**DOI:** 10.3390/diagnostics12081890

**Published:** 2022-08-04

**Authors:** Fabrizia Gelardi, Elisa Maria Ragaini, Martina Sollini, Daniela Bernardi, Arturo Chiti

**Affiliations:** 1 Department of Biomedical Sciences, Humanitas University, Via Rita Levi Montalcini 4, 20072 Pieve Emanuele, Italy; 2 IRCCS Humanitas Research Hospital, Via Manzoni 56, 20089 Rozzano, Italy

**Keywords:** breast cancer, contrast-enhanced mammography, contrast-enhanced breast magnetic resonance imaging, screening

## Abstract

Background: Contrast-enhanced mammography (CEM) and contrast-enhanced magnetic resonance imaging (CE-MRI) are commonly used in the screening of breast cancer. The present systematic review aimed to summarize, critically analyse, and meta-analyse the available evidence regarding the role of CE-MRI and CEM in the early detection, diagnosis, and preoperative assessment of breast cancer. Methods: The search was performed on PubMed, Google Scholar, and Web of Science on 28 July 2021 using the following terms “breast cancer”, “preoperative staging”, “contrast-enhanced mammography”, “contrast-enhanced spectral mammography”, “contrast enhanced digital mammography”, “contrast-enhanced breast magnetic resonance imaging” “CEM”, “CESM”, “CEDM”, and “CE-MRI”. We selected only those papers comparing the clinical efficacy of CEM and CE-MRI. The study quality was assessed using the QUADAS-2 criteria. The pooled sensitivities and specificity of CEM and CE-MRI were computed using a random-effects model directly from the STATA “metaprop” command. The between-study statistical heterogeneity was tested (I^2^-statistics). Results: Nineteen studies were selected for this systematic review. Fifteen studies (1315 patients) were included in the metanalysis. Both CEM and CE-MRI detect breast lesions with a high sensitivity, without a significant difference in performance (97% and 96%, respectively). Conclusions: Our findings confirm the potential of CEM as a supplemental screening imaging modality, even for intermediate-risk women, including females with dense breasts and a history of breast cancer.

## 1. Introduction

Breast cancer is the malignancy with the highest incidence worldwide, with an estimated 2.3 million new cases in 2020, representing a significant health burden [1]. For this reason, many efforts are being made to try to identify new techniques that could help in the early detection, diagnosis, and preoperative assessment of such tumours. As the early detection of breast cancer is an important and frequent subject of debate in the healthcare system, many techniques have been developed over time for screening purposes [2]. In the 20th century, mammography (MG) was the chosen method to investigate breast lesions; however, it was not until the 1980s that this technique was also considered for screening. During these years in fact, nine randomized controlled trials (RCTs) were carried out investigating the benefits of screening using MG. These studies demonstrated that screening with mammography resulted in the detection of small lesions, leading to favourable outcomes [3]. Through the years, MG has been improved and modernized, and nowadays, digital MG represents one of the most valid secondary prevention techniques for breast cancer, along with digital breast tomosynthesis (DBT), which is becoming a viable alternative, especially for clinical use [4]. Breast ultrasound (US) is frequently used as a supplementary tool in patients that have a reduced sensitivity to MG, for example, in the case of dense breasts [5]. Although it can be useful in these situations, US is often penalized because of its low specificity [6]. Another technique frequently used in the screening of breast cancer is magnetic resonance imaging (MRI), which holds the highest sensitivity for the detection of occult cancer [7]. MRI is recommended for the screening of woman at high-risk of breast cancer, and it may be considered in cased of intermediate risk, including in females with dense breasts and a history of breast cancer [8]. Finally, nuclear medicine techniques can also be used, such as ^99m^TC-MIBI for scintigraphy and 2-Deoxy-2-[18F] fluoroglucose ([18F] FDG) for PET/CT. ^99m^TC-MIBI is not widely used to image breast cancers because of the whole-body radiation dose. Lastly, breast PET exploits [18F] FDG uptake to image cancer cells; therefore, even if it can be theoretically used for screening purposes, its use remains limited because of radiation exposure and high costs [4]. Contrast-enhanced methodologies, namely contrast-enhanced mammography (CEM) and contrast-enhanced magnetic resonance imaging (CE-MRI), are nowadays commonly used in the screening of breast cancer, as they allow for depicting the neo-vasculature brought about by the tumour, providing functional information. CEM is a dual-energy imaging technique that uses an iodinated contrast agent to enhance tumoral vessels, allowing for the detection of breast morphological abnormalities, while being relatively cheap and quick [9,10,11]. CE-MRI, instead, uses a gadolinium-based contrast that accumulates into the stroma of the cancer. The collection of the contrast agent in this specific location occurs because the newly formed vessels, necessary for the tumour to grow, are leaky and therefore allow for the extravasation of such a contrast agent into the tumoral stroma [12]. Because of the aforementioned mechanisms, both CE-MRI and CEM exploit tumour angiogenesis to detect breast lesions, and because of their ability to enhance tumoral vessels, they are good candidates to increase the performance of MG in its different uses [7,10,11,13].

The present systematic review aimed to summarize and critically analyse the available evidence about the role of CE-MRI and CEM in the early detection, diagnosis, and preoperative assessment of breast cancer. Secondary, we meta-analysed the diagnostic performance of both CE-MRI and CEM in the early detection, diagnosis, and preoperative assessment of breast cancer.

## 2. Materials and Methods

### 2.1. Literature Search and Study Selection

The systematic review was carried out following the PRISMA statement. A two-step search and evaluation strategy was adopted and executed independently by one reviewer (ER). The first step consisted in selecting studies present on PubMed by using the following keywords: “breast cancer”, “preoperative staging”, “contrast-enhanced mammography”, “contrast-enhanced spectral mammography”, “contrast enhanced digital mammography”, “contrast-enhanced breast magnetic resonance imaging”, “CEM”, “CESM”, “CEDM”, and “CE-MRI”. Secondly, the Google Scholar database was searched, using the aforementioned terms, and the resulting matching manuscripts were listed in an excel file. The search was performed by two researchers (M.S. and F.G.) on 28 July 2021, and no starting date was applied. For the article selection, the list was first screened for duplicates, which were removed, and then screened to identify only those papers comparing the clinical efficacy of the contrast-enhanced mammography vs. contrast-enhanced breast magnetic resonance. The inclusion and exclusion criteria were defined. The inclusion criteria were as follows: (a) full-text available, (b) only manuscripts comparing the two methods (CEM and CE-MRI), and (c) full text available in the English language. The exclusion criteria were as follows: (a) out of the scope of the present review and meta-analysis; (b) preclinical studies without translational aspects (i.e., not involving human subjects); (c) phantom, analytical, or simulation studies; (d) case report and case series (less than 10 patients); (e) editorials, commentaries, and reviews; and (f) conference proceedings. The titles and abstracts of the articles identified were reviewed applying the aforementioned inclusion/exclusion criteria, and full-text versions of the selected articles were downloaded.

### 2.2. Quality Assessment

The quality of each study was assessed independently by two reviewers (E.R. and M.S.) using the Quality Assessment of Diagnostic Accuracy Studies 2 (QUADAS-2) criteria. For each study, the risk of bias and the applicability of the primary diagnostic accuracy studies were evaluated, and for both domains (risk of bias and applicability) we assigned qualitative measures indicated as “unclear”, “low”, or “high”. We assigned 0.5 points in the case of an “unclear” score, 1 point in the case of “high risk of bias/low applicability”, and “zero” in the case of “low risk of bias/high applicability”. Discordant results were discussed, and discrepancy was solved by consensus. Lastly, the cumulative scores were calculated. Articles with a cumulative score higher than 3 were considered ineligible, and they were excluded from the subsequent analysis.

### 2.3. Data Collection

For each study, we collected the following information: general features (title, name of the authors, and year of publication) (1); aim of the study (2); study design (3); number of patients (4); number of lesions (5); reference standard used as the final diagnosis (6); accuracy of CE-MRI and CEM (7); sensitivity of CE-MRI and CEM, and specificity of CE-MRI and CEM (8); positive predictive value of CE-MRI and CEM, and negative predictive value of CE-MRI and CEM (9); AUC of CE-MRI and CEM (10); true positive/negative and false positive/negative results (11); dose of contrast medium (12); acquisition time (13); and the main results from the study (14). In the case of missing data, this was requested from the corresponding author by email.

### 2.4. Meta-Analysis

In the meta-analysis, we included only studies providing complete data to build a contingency table for both CEM and MRI. We performed a per-lesion analysis, equally considering the index and secondary lesions and merging the data to build a single contingency table. In studies comparing diagnostic ability through the interpretation of more than one radiologist, the results with the highest sensitivity were considered for the analysis. The sensitivity, specificity, and 95% confidence intervals (CIs) for both CEM and CE-MRI were calculated from each study. Forest plots of the estimated pooled sensitivities and specificities (with 95% confidence intervals) were built. The weight assigned to each study was computed from STATA with a random effects model by running the “metaprop” command. The Freeman–Tukey double arcsine transformation was performed to stabilize the variances before pooling [14]. Between-study statistical heterogeneity was estimated to evaluate the data consistency using I^2^ and Cochran’s Q homogeneity test. We scored the heterogeneity as low, moderate, or high. A low/moderate level of heterogeneity (i.e., I^2^ < 75%) was identified as reliable, while we considered sub-analyses in the case of high heterogeneity among studies [15]. Publication and other potential bias were assessed with funnel plots. The Egger method was applied to test the funnel plot asymmetry. A *p*-value ≤ 0.05 was considered for statistical significance. Statistical analyses were performed using STATA (STATA version 17.0 StataCorp LP, College Station, TX, USA).

## 3. Results

### 3.1. Study Selection

The first search on PubMed using the above keywords produced 870 results. Subsequently, 750 papers were excluded based on their title and abstract. Among the 120 articles we found, 17 compared the value of both CEM and CE-MRI. Subsequently, after searching on Google Scholar, one other paper that was not previously found was selected. No other articles were found on Web of Science. From a meta-analysis comparing the clinical efficacy of CEM and CE-MRI [13], we identified five other articles. Among these, one was excluded because the full text was not in the English language, and three others were excluded because only the abstracts had been published within scientific meetings and conferences. Nineteen articles were assessed for quality using the QUADAS2 score and were included in the systematic analysis.

### 3.2. Systematic Review

**Figure 1:**
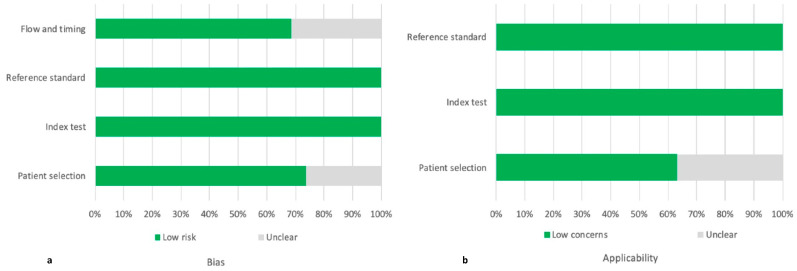
Assessment of the risk of bias (**a**) and the applicability (**b**) through the QUADAS-2 score.

The QUADAS-2 score identified concerns about patient selection and flow and timing in 5/19 and 6/19 studies, respectively (Figure 1). Nonetheless, the cumulative QUADAS-2 score of the 19 studies was acceptable; therefore, they were all included in the analysis (Supplementary File S1).

Rudnicki et al. [16] recently compared MRI and CEM in 121 patients with dense breast and abnormalities detected upon ultrasound (US) or mammography (MG). The CEM accuracy and specificity were higher (77% vs. 74 and 33% vs. 23%, respectively) compared with MRI in this cohort of females with dense breasts. Luczynska et al. [17] focused on evaluation of the background parenchymal enhancement (BPE) in addition to the qualitative assessment as a potential method to add value to both CEM and MRI. They analysed 64 patients. Strong BPE was more frequent among malignant lesions than benign ones for CEM, providing added value to the sole qualitative description of the lesion enhancement level.

In a prospective single centre study, Clauser et al. [18] compared the diagnostic performance of low radiation dose contrast-enhanced mammography (L-CEM) to CE-MRI. While the sensitivity was slightly higher in CE-MRI than in L-CEM (83.6–93.4% vs. 65.6–90.2%), both the specificity and positive predictive value (PPV) were higher in L-CEM than in CE-MRI (46.9–96.9% and 76.4–97.6% vs. 37.5–53.1% and 73.3–77.3%).

Cheung et al. [19] compared the effectiveness of CEM to CE-MRI in the preoperative detection of 51 breast lesions diagnosed upon sonographic-guided biopsy. CEM was successful for the malignancy extension assessment in patients with diagnosed breast cancers. Marino et al. [20] investigated the value of the radiomic analysis of CEM and CE-MRI on 49 surgically confirmed breast lesions, demonstrating a strong correlation with the tumour histology, hormone receptor status, and tumour grade.

Xing et al. [21] showed an equal sensitivity for both CEM and CE-MRI (91.5%) when retrospectively analysing 235 patients. The specificity and accuracy of CEM were higher than CE-MRI (89.5% vs. 80.2% and 81% vs. 71.7%, respectively), with a lower false-positive rate. Sumkin et al. [22] prospectively investigated 102 patients with surgically proven breast lesions using CE-MRI, CEM, and molecular breast imaging (MBI), showing a similar detection rate (93%, 91%, and 92%, respectively). Youn et al. [23] demonstrated that CEDM and CE-MRI have a similar accuracy when measuring breast tumour size. Kim et al. [24] observed that the sensitivity of CEDM was slightly lower than for CE-MRI (92.2% vs. 95.2%), while the specificity, accuracy, PPV, and NPV of CEDM were higher (81.1%, 82.1%, 94.7%, and 83.7% vs. 73.6%, 77.4%, 90.5%, and 82.1%, respectively). Additionally, the percentage of false-positives was lower in CEDM than in CE-MRI (10.5% vs. 19.8%).

Li et al. [25] showed a comparable sensitivity but a slightly higher accuracy and PPV for CEM than for CE-MRI (96.9% vs. 93.9% and 97% vs. 94%, respectively). They also highlighted that CEM had a significantly lower BPE. Lee-Felker et al. [26] compared the diagnostic performances of CEM and CE-MRI for the detection of index and secondary breast cancers. The sensitivity was higher in CE-MRI than in CEM (99% vs. 94%), while the specificity and PPV were lower (4% and 17% vs. 60% and 93%, respectively). The authors concluded that CEM is possibly as valid as CE-MRI in the assessment of newly diagnosed breast cancer. Fallenberg et al. [27] demonstrated a lower sensitivity than previous studies both for CE-MRI and CEM (76% and 72%, respectively). Knogler et al. [28] demonstrated a comparable effectiveness for CEDEM and CE-MRI when analysing 11 breast lesions over a 9-month period. Wang et al. [29] evaluated the performance of CE-MRI compared with CEM, achieving a higher specificity, accuracy, PPV, and NPV (82.8%, 89.6%, 82.2%, and 92.3% vs. 65.5%, 84.4%, 82.1%, and 90.5%, respectively) and a lower sensitivity (93.8% vs. 95.8%) in contrast with most of the previous works.

Chou et al. [30] compared the diagnostic accuracy of CEDM, contrast-enhanced tomosynthesis (CET), and CE-MRI, which showed a similar AUC. Moreover, the diagnostic performance of the contrast-enhanced aforementioned tools was significantly higher compared with the conventional non-enhanced modalities. Łuczyńska et al. [31] found that the sensitivity, accuracy, PPV, and NPV of the CEM were substantially higher compared with CE-MRI (100%, 79%, 77%, and 100% vs. 93%, 73%, 74%, and 65%, respectively). Similarly, Lobbes et al. [32] compared the diagnostic value of CEM and CE-MRI and came to the conclusion that the former is a feasible imaging modality for the detection of breast cancer. Fallenberg et al. [33] compared MG, CEM, and CE-MRI for the detection and size assessment of histologically proven lesions, observing a superior sensitivity for CEM and CE-MRI with respect to MG. Moreover, CEM showed a higher sensitivity than CE-MRI (100% vs. 97.4%). Finally, Jochelson et al. [34] showed a comparable detection rate for known primary tumours for CEDM and CE-MRI.

### 3.3. Meta-Analysis

Four articles not providing complete data to build a contingency table were excluded from the quantitative analysis. Finally, 15 studies on 1315 patients were included (Supplementary Materials).

**Figure 2:**
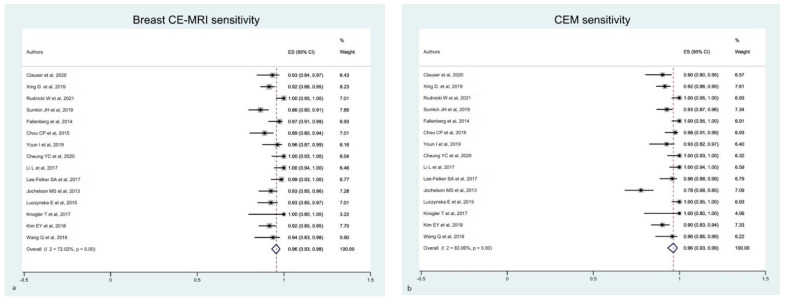
Forest plot of the estimated pooled sensitivity of breast CE-MRI (**a**) and CME (**b**) in the detection of pathological breast lesions, including index and secondary lesions. The estimated pooled sensitivity of CE-MRI (**a**) was 0.96 (95% CI 0.93–0.98), with a moderate heterogeneity (I^2^ = 72.02% *p* = 0.001). The estimated pooled sensitivity of CME (**b**) was 0.96 (95% CI 0.93–0.99), with a high heterogeneity (I^2^ = 83.06% *p* = 0.001) [16,17,18,19,21,22,23,24,25,26,28,29,30,33,34].

The estimated pooled sensitivity of breast CE-MRI and CEM was comparable, although studies on CEM presented a higher heterogeneity than CE-MRI (Figure 2).

**Figure 3:**
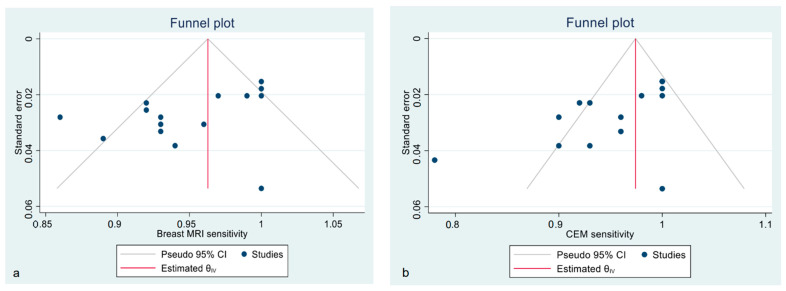
Funnel plot with 95% CIs for publication bias assessment of pooled sensitivity of CE-MRI (**a**) and CEM (**b**) in the detection of pathological breast lesions (considering both index and secondary lesions).

Funnel plots show the asymmetrical distribution of dots in both the CE-MRI and CEM sensitivity analyses (Figure 3), confirmed by the Egger regression-based test for funnel plot asymmetry (bias −2.45, SE 1.229, *p* = 0.046 for CE-MRI; bias −3.49, SE 0.081, *p* < 0.001 for CEM). The estimated pooled specificity was higher for CEM than CE-MRI; however, all of the included studies presented a high heterogeneity, regardless of the technique (Supplementary Figure S1).

**Figure 4:**
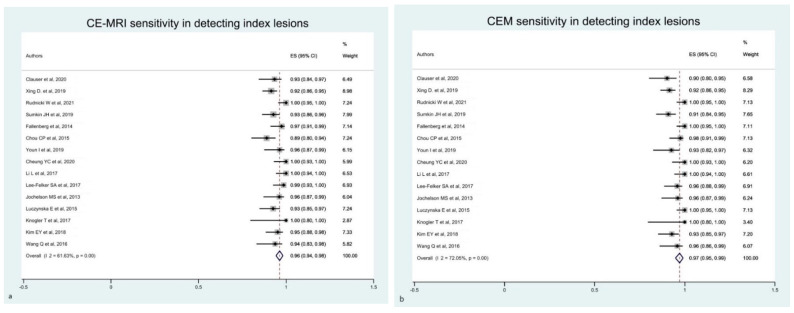
Forest plot of the estimated pooled sensitivity of breast CE-MRI (**a**) and CME (**b**) in the detection of pathological breast index lesions. The estimated pooled sensitivity of CE-MRI (**a**) was 0.96 (95% CI 0.94–0.98), with a moderate heterogeneity (I^2^ = 61.63% *p* = 0.001). Estimated pooled sensitivity of CME (**b**) was 0.97 (95% CI 0.95–0.99), with a moderate heterogeneity (I^2^ = 72.05% *p* = 0.001) [16,17,18,19,21,22,23,24,25,26,28,29,30,33,34].

In light of the high between-study heterogeneity, we performed additional sub-analyses. Firstly, we considered only the CEM and CE-MRI ability for diagnosing only index lesions. The estimated pooled sensitivity of CEM was slightly improved compared with the previous analysis (i.e., primary analysis), with a reduction in heterogeneity (Figure 4a). We observed a similar reduction in heterogeneity also considering CE-MRI, although without a significant difference in sensitivity (Figure 4b).

**Figure 5:**
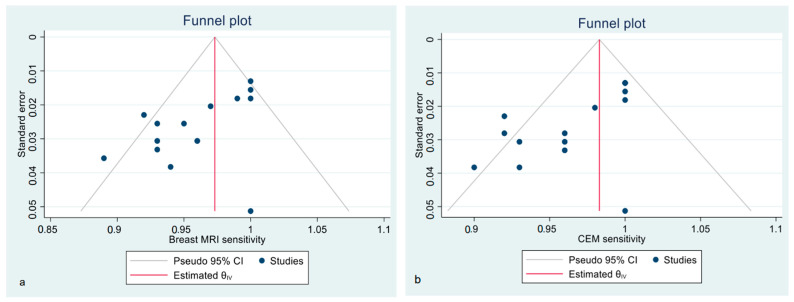
Funnel plot with 95% CIs for publication bias assessment of the pooled sensitivity of CE-MRI (**a**) and CEM (**b**) for the detection of pathological breast index lesions.

The estimated pooled specificity of CE-MRI and CEM for diagnosing index lesions was low, along with a persistent high heterogeneity (Supplementary Figure S2). Funnel plots showed an asymmetrical distribution of dots in both the CE-MRI and CEM sensitivity analysis (Figure 5), confirmed by the Egger regression-based test for funnel plot asymmetry (bias −2.90, SE 0.75, *p* = 0.001 for MRI; bias −2.78, SE 0.66, *p* < 0.001 for CEM).

**Figure 6:**
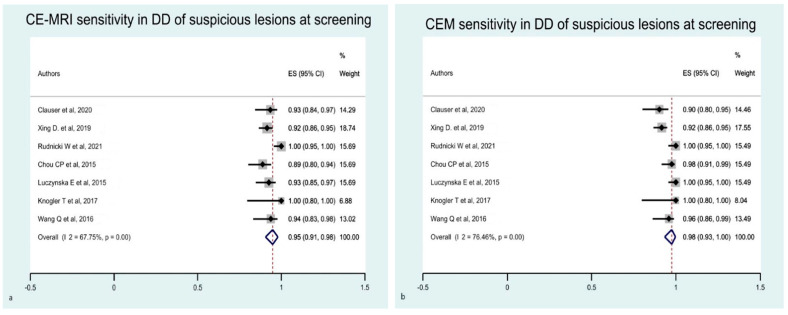
Forest plot of the estimated pooled sensitivity of breast CE-MRI (**a**) and CME (**b**) for the differential diagnosis of suspicious lesions at screening. The estimated pooled sensitivity of CE-MRI (**a**) was 0.95 (95% CI 0.91–0.98), with a moderate heterogeneity (I^2^ = 67.75% *p* = 0.001). The estimated pooled sensitivity of CME (**b**) was 0.98 (95% CI 0.93–1.00), with a high heterogeneity (I^2^ = 76.46% *p* = 0.001) [16,17,18,21,28,29,30].

Secondly, we evaluated the diagnostic performance of the two techniques based on clinical indication and target population differential diagnosis of suspicious lesions at screening and preoperative staging, respectively. We considered only index lesions in these analyses. For the differential diagnosis of suspicious lesions at screening, the estimated pooled sensitivity of CEM was slightly improved compared with the principal analysis, while the values for CE-MRI were slightly worse (Figure 6). The estimated pooled specificity of CE-MRI and CEM for differentiating suspicious lesions at screening was improved compared with previous analysis, but the studies were burdened by a high heterogeneity (Supplementary Figure S3).

**Figure 7:**
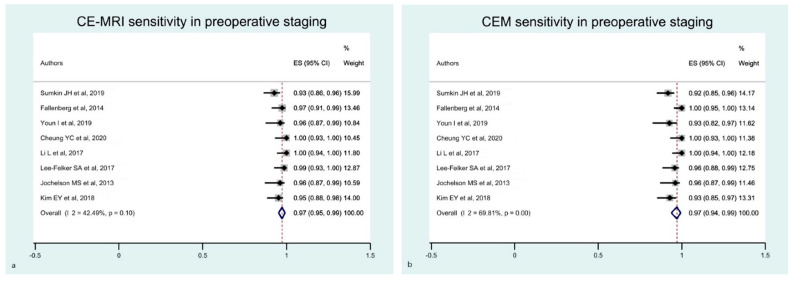
Forest plot of the estimated pooled sensitivity of breast CE-MRI (**a**) and CME (**b**) in preoperative staging. The estimated pooled sensitivity of CE-MRI (**a**) was 0.97 (95% CI 0.95–0.99), with a moderate heterogeneity (I^2^ = 42.49% *p* = 0.01). The estimated pooled sensitivity of CME (**b**) was 0.97 (95% CI 0.94–0.99), with a moderate heterogeneity (I^2^ = 69.81% *p* = 0.10) [19,22,23,24,25,26,33,34].

**Figure 8:**
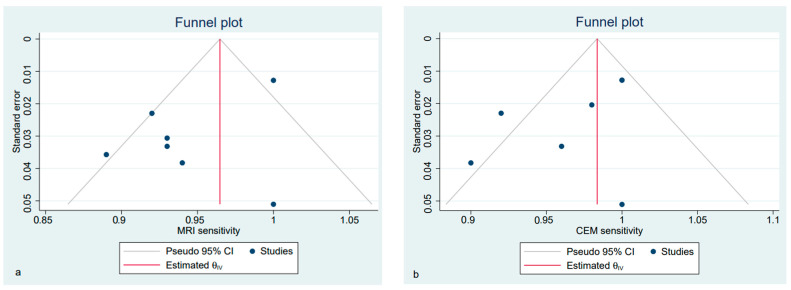
Funnel plot with 95% CIs for publication bias assessment of the pooled sensitivity of CE-MRI (**a**) and CEM (**b**) for the differential diagnosis of suspicious lesions at screening.

**Figure 9:**
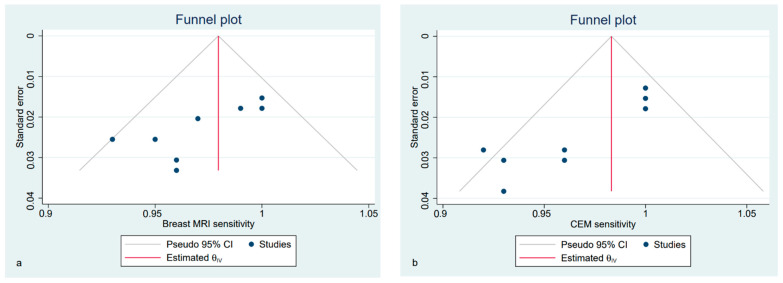
Funnel plot with 95% CIs for publication bias assessment for the pooled sensitivity of CE-MRI (**a**) and CEM (**b**) in preoperative staging.

For the preoperative staging, the estimated pooled sensitivity of breast CE-MRI and CEM were comparable (Figure 7). The funnel plots showed an asymmetrical distribution of dots in all of these analyses (Figure 8 and Figure 9, respectively). The estimated pooled specificity of CE-MRI and CEM was very low (Supplementary Figure S4).

**Figure 10:**
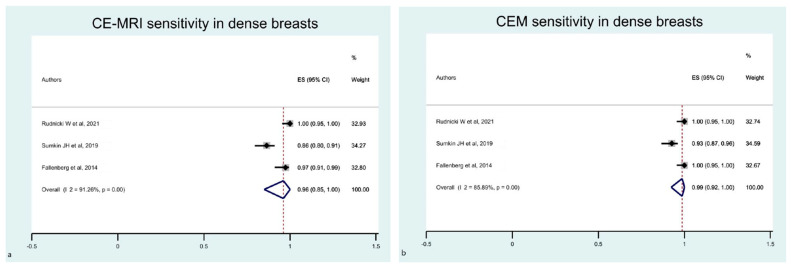
Forest plot of the estimated pooled sensitivity of breast CE-MRI (**a**) and CME (**b**) in the detection of pathological breast lesions in women with dense breasts. The estimated pooled sensitivity of CE-MRI was 0.98 (95% CI 0.91–1.00; I^2^ = 78.96% *p* = 0.001). The estimated pooled sensitivity of CEM was 0.99 (95% CI 0.92–1.00;I^2^= 85.89% *p* = 0.001) [16,22,33].

**Figure 11:**
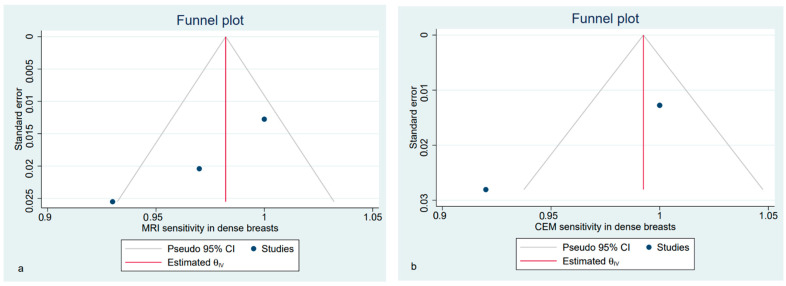
Funnel plot with 95% CIs for publication bias assessment of pooled sensitivity for CE-MRI (**a**) and CEM (**b**) for the detection of pathological breast lesions in women with dense breasts.

Lastly, we focused only on dense breasts, including in the sub-analysis only studies enrolling more than 50% women with dense breasts in the study cohort. We identified five eligible studies, two of which were excluded because of the lack of complete data. Therefore, three papers were analysed only for sensitivity. The estimated pooled sensitivity of breast CE-MRI and CEM was higher than in previous analyses (Figure 10). Funnel plots showed the asymmetrical distribution of dots in both the MRI and CEM sensitivity analyses (Figure 11).

Table 1 summarizes the results (i.e., sensibility, specificity, heterogeneity, and significance) of all of the analyses performed.

## 4. Discussion

Our results confirm that CEM and CE-MRI could be reliable for screening, as they are successful in the detection, diagnosis, and preoperative assessment of breast cancer, even in patients with dense breasts. We conclude that CEM and CE-MRI are both valid options with a high sensitivity for diagnosing breast cancer. Specifically, our meta-analysis shows that both CEM and CE-MRI detected breast lesions with a high sensitivity (both 0.96) (Table 1). Nonetheless, CEM performed slightly better than CE-MRI in some circumstances, such as for detecting index lesions (0.97 vs. 0.96), differentiating suspicious lesions at screening (0.98 vs. 0.95), and in dense breasts (0.99 vs. 0.98), becoming a reliable alternative to CE-MRI.

CE-MRI exhibited a lower specificity compared with CEM for diagnosing breast lesions, mainly related to the high number of false positives encountered among the secondary lesions [16,18,21,24,25,26,31,33], as confirmed by our meta-analysis (0.30 vs. 0.43, respectively).

CEM has a better performance for the evaluation of suspicious calcifications compared with CE-MRI and offers several practical advantages. CE-MRI is time-consuming, expensive, not widely accessible, and not feasible in patients with non-MR compatible cardiac devices or claustrophobia. On the other hand, MRI does not require exposure to radiation and breast compression [3,4,35].

Our results are only partially reliable because of the moderate to high heterogeneity found among the studies. Between-study heterogeneity may be explained by different design and methodological approaches, as well as the great variability in terms of the sample size and number of lesions analysed (Supplementary File S1). Nonetheless, our results are consistent with those of a previous publication by Xiang et al. [13], although the studies included in the meta-analysis differed. Although they reported a higher estimated pooled specificity compared with our results in terms of absolute numbers, the improvement in terms of the performance of CEM over CE-MRI was almost reproducible, with an increase of 14% (66% vs. 52%) and 13% (43% vs. 30%), respectively, and a high between-study heterogeneity confirmed. Notably, between-study heterogeneity improved when we considered only the index lesions. Nonetheless, the success gained was limited, as it was related to a slight decrease in heterogeneity from high to moderate in the sensitivity analysis, while we did not observe any significant changes in the specificity analysis. Compared with the previously published meta-analyses, we assessed the performance of the two imaging tools in different clinical settings, including the differential diagnosis of suspicious lesions at screening, preoperative staging, and diagnosis in patients with dense breast tissue.

The lower sensitivity of conventional MG when screening patients with dense breasts has resulted in great efforts to identify the imaging tool with a higher performance. A higher sensitivity has been demonstrated by both MRI and CEM compared with MG alone, whereas MRI lacked specificity, resulting in a high number of false positives [8,36]. From our results, only three studies performed a head-to-head comparison of the two methods in this sub-group of patients, confirming the high sensitivity of both MRI and CEM. Unfortunately, no conclusion can be drawn in terms of specificity. However, the current guidelines do not justify the use of MRI for screening purposes in asymptomatic patients with no suspicious alterations at screening [37,38].

Unenhanced MRI protocols, including diffusion-weighted imaging (DWI) and diffusion tensor imaging (DTI), are emerging to improve the diagnostic ability of breast MRI [39]. DWI and DTI protocols showed a high ability to discriminate benign and malignant breast lesions, with a higher specificity compared with CE-MRI, thus decreasing the use of unnecessary biopsies. However, the absence of standardized protocols, high costs, and image quality issues prevent the widespread use of unenhanced MRI techniques [40,41,42].

Our study has several limitations. The low number of the eligible studies, particularly in the sub-analyses in different clinical settings, might have affected results. Moreover, the study designs and sample sizes varied widely between the different included studies, as evidenced by the relatively high heterogeneity. We harmonized the studies by performing different sub-analyses, but this strategy furtherly reduced the sample size. Lastly, most studies were performed by evaluating patients with already known primary lesions or suspected lesions at screening, which might have impacted the sensitivity and specificity.

## 5. Conclusions

Our findings confirm the potential of CEM as a supplemental screening imaging modality, even for intermediate-risk women, including females with dense breasts and a history of breast cancer. Both CE-MRI and CEM exhibit a high sensitivity, whereas CE-MRI exhibits a lower specificity compared with CEM for diagnosing breast lesions. The lower specificity of CE-MRI mainly related to the high number of false positives encountered among the secondary lesions might result in a high number of unnecessary invasive biopsies. CEM is a viable screening method because of its lower costs, higher availability, shorter acquisition times, and higher patient tolerance.

## Figures and Tables

**Table 1:** The results of all of the analyses performed.

Principal Analyses		Sensitivity (95% CI)	Heterogeneity; *p*-Value	Specificity (95% CI)	Heterogeneity; *p*-Value
*All detected lesions*	**CE-MRI**	0.96 (CI 0.93–0.98)	I^2^ 72.02%; *p* = 0.001	0.30 (CI 0.11–0.52)	I^2^ **93.90%**; *p* = 0.001
	**CEM**	0.96 (CI 0.93–0.99)	I^2^ **83.06%**; *p* = 0.001	0.43 (CI 0.25–0.63)	I^2^ **88.01%**; *p* = 0.001
**Secondary analyses**					
*Index lesions detection*	**CE-MRI**	0.96 (CI 0.94–0.98)	I^2^ 61.63%; *p* = 0.001	0.35 (CI 0.13–0.61)	I^2^ **94.31%**; *p* = 0.001
	**CEM**	0.97 (CI 0.95–0.99)	I^2^ 72.05%; *p* = 0.001	0.38 (CI 0.17–0.61)	I^2^ **90.68%**; *p* = 0.001
DD of suspicious lesions at screening *	**CE-MRI**	0.95 (CI 0.91–0.98)	I^2^ 67.75%; *p* = 0.001	0.55 (CI 0.26–0.82)	I^2^ **94.06%**; *p* = 0.001
	**CEM**	0.98 (CI 0.93–1.00)	I^2^ **76.46%**; *p* = 0.001	0.58 (CI 0.32–0.82)	I^2^ **92.63%**; *p* = 0.001
Pre-operative staging *	**CE-MRI**	0.97 (CI 0.95–0.99)	I^2^ 42.49%; *p* = 0.10	0.08 (CI 0.0–0.23)	I^2^ 73.60%; *p* = 0.001
	**CEM**	0.97 (CI 0.94–0.99)	I^2^ 69.81%; *p* = 0.001	0.27 (CI 0.02–0.62)	I^2^ **80.91%**; *p* = 0.001
*Diagnosis in dense breasts*	**CE-MRI**	0.98 (CI 0.91–1.00)	I^2^ **78.96%**; *p* = 0.01	NA	NA
	**CEM**	0.99 (CI 0.92–1.00)	I^2^ **85.89%**; *p* = 0.001	NA	NA

CI: confidence interval; DD: differential diagnosis; NA: not assessed; value of I^2^ in bold = high heterogeneity (more than 75%); * analysis performed only considering the index lesion.

## Data Availability

The manuscript represents valid work, and neither this manuscript nor one with substantially similar content under the same authorship has been published or is being considered for publication elsewhere.

## Supplementary Materials

The following supporting information can be downloaded at: https://www.mdpi.com/article/10.3390/diagnostics12081890/s1.
