# Supplementary figures and images for: Contrast-Enhanced Mammography versus Breast Magnetic Resonance Imaging: A Systematic Review and Meta-Analysis
Source: Diagnostics (Basel). 2022 Aug 4;12(8):1890. doi: 10.3390/diagnostics12081890 (PMC9406751; doi:10.3390/diagnostics12081890)

# Breast MRI specificity

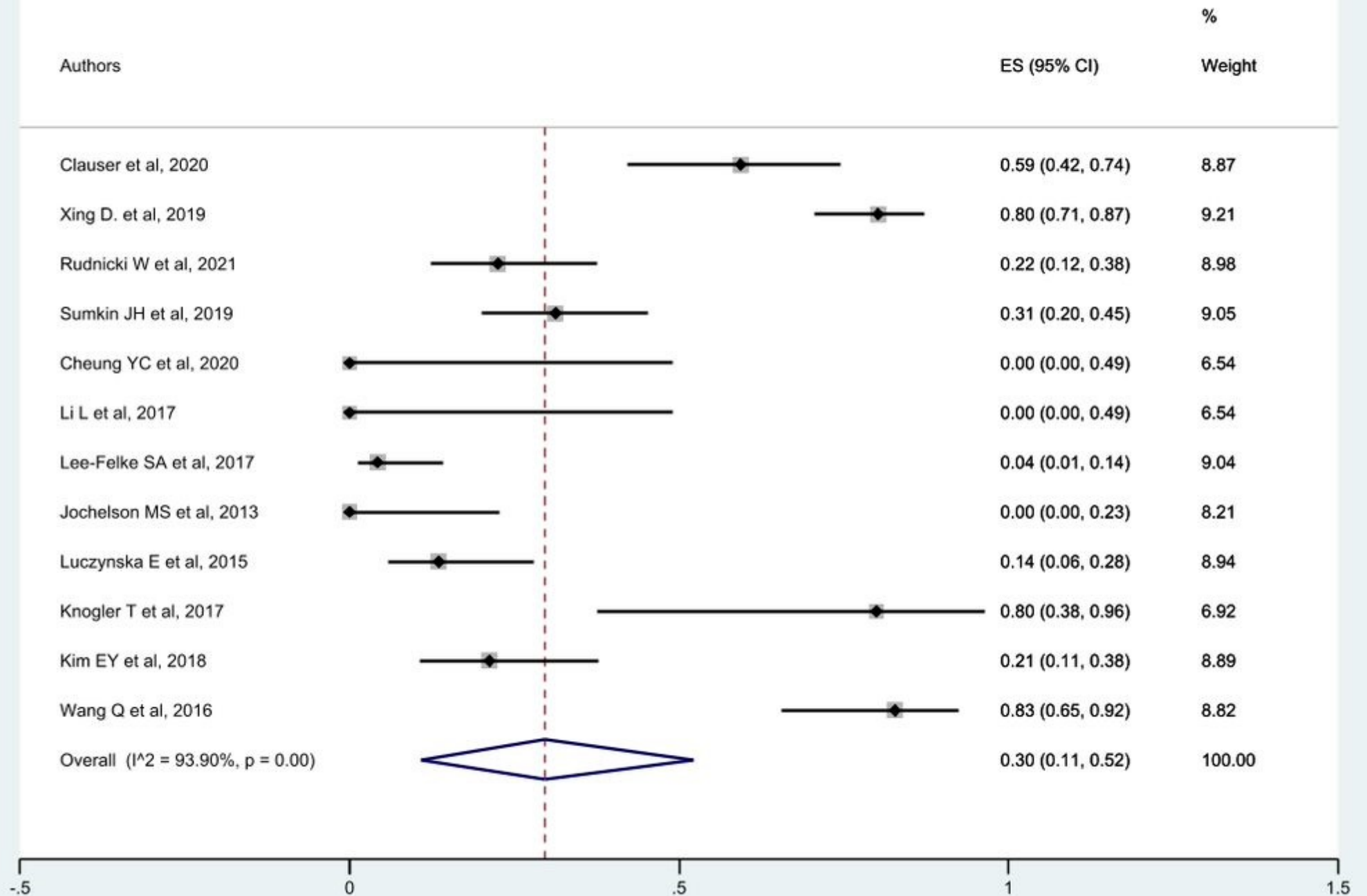

Supplement: Supplementary file 1 [file diagnostics-12-01890-s001.zip › Figure S1a.pdf]

# CEM specificity

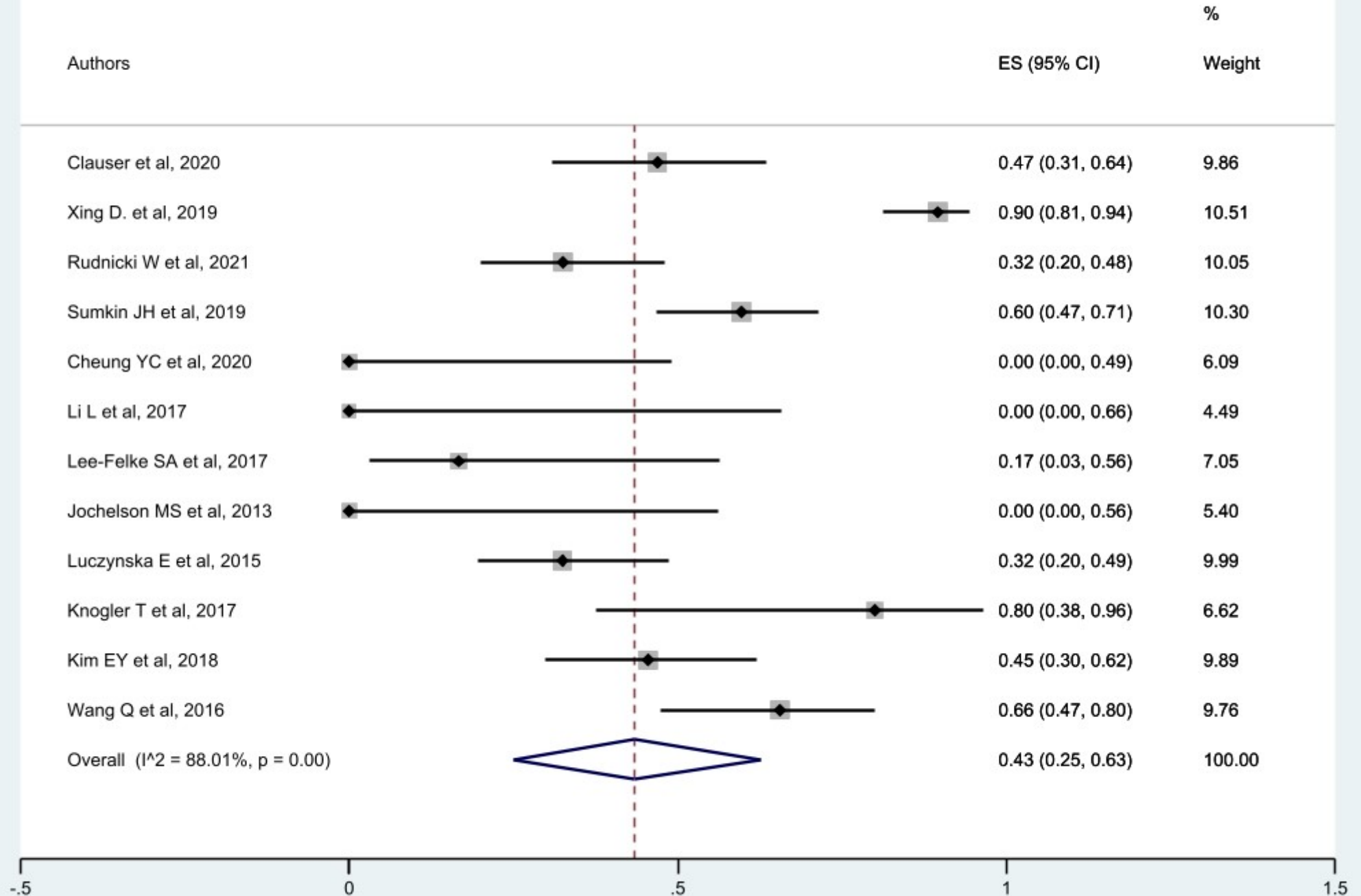

Supplement: Supplementary file 1 [file diagnostics-12-01890-s001.zip › Figure S1b.pdf]

# Breast MRI specificity

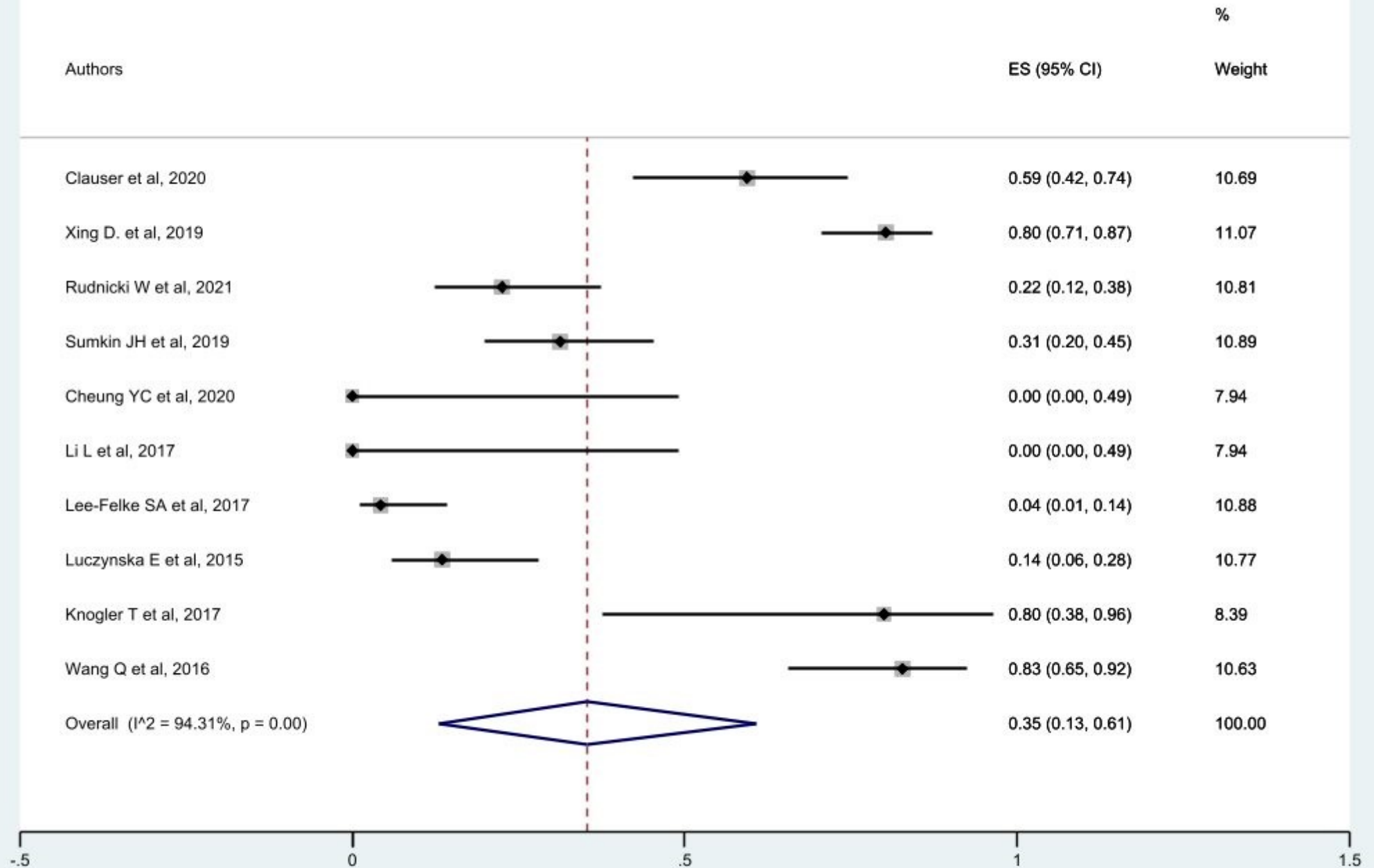

Supplement: Supplementary file 1 [file diagnostics-12-01890-s001.zip › Figure S2a.pdf]

# CEM specificity

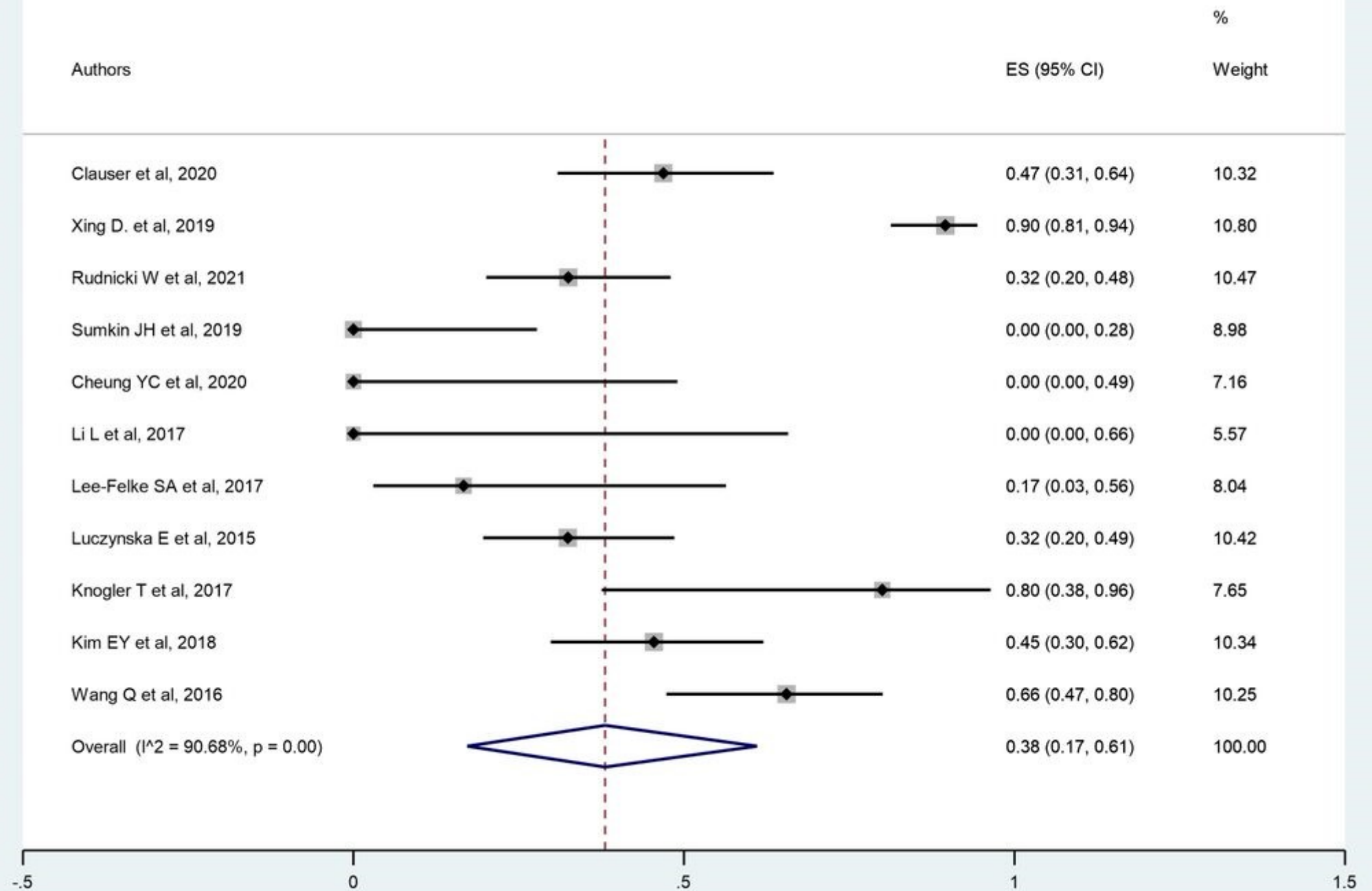

Supplement: Supplementary file 1 [file diagnostics-12-01890-s001.zip › Figure S2b.pdf]

# Breast MRI specificity for differential diagnosis of suspicious lesions

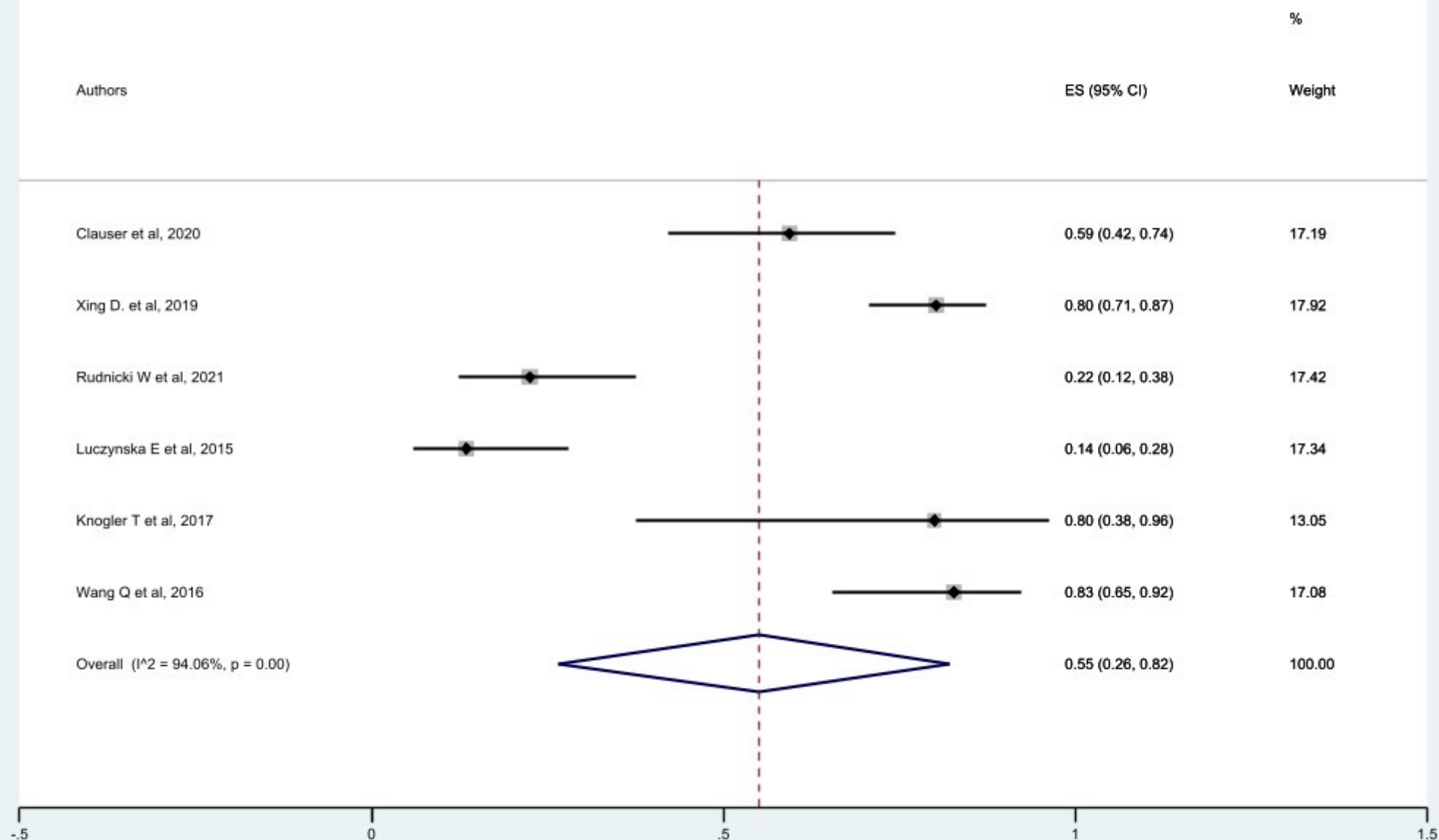

Supplement: Supplementary file 1 [file diagnostics-12-01890-s001.zip › Figure S3a.pdf]

## Breast CEM specificity for differential diagnosis of suspicious lesions

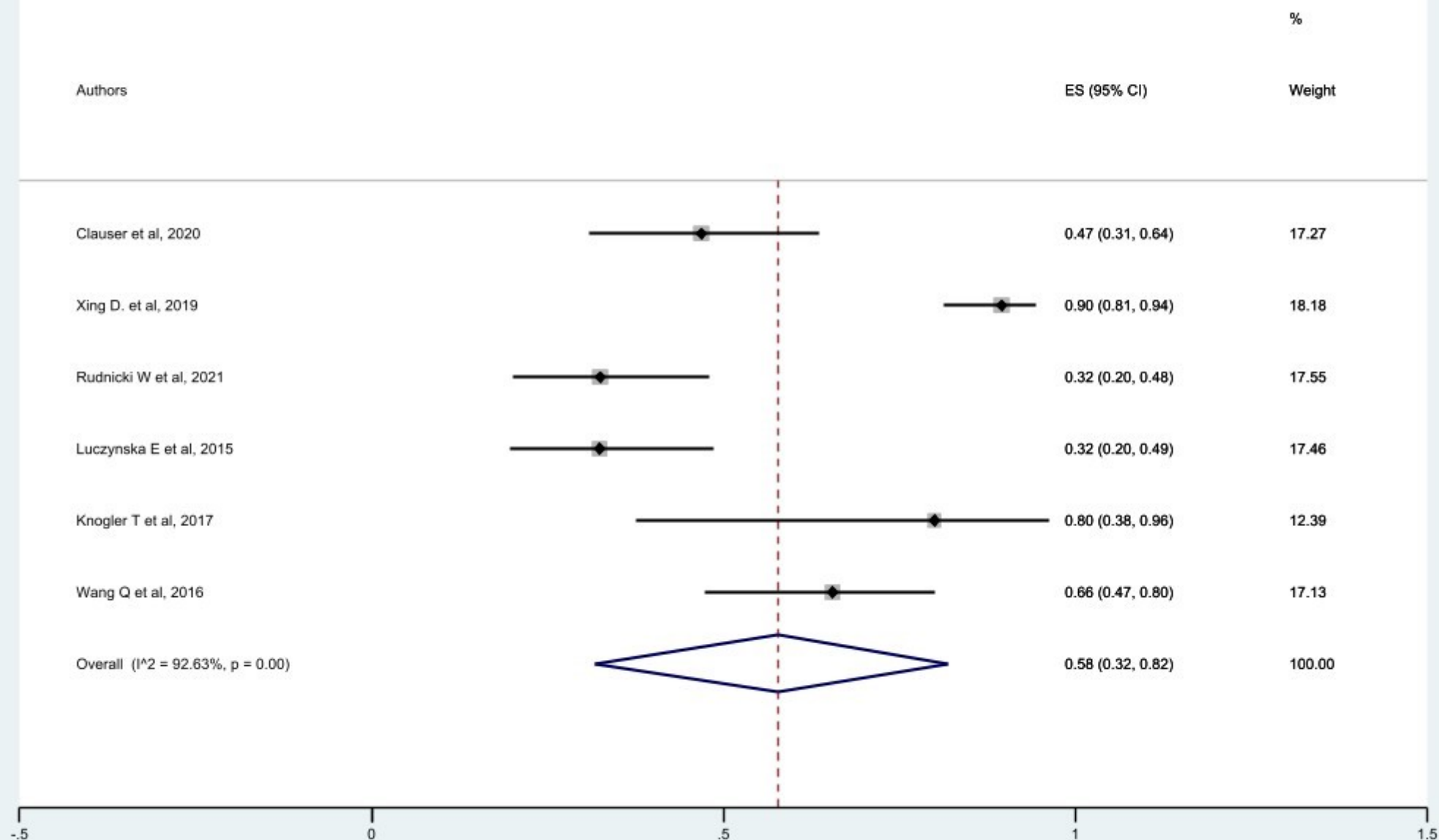

Supplement: Supplementary file 1 [file diagnostics-12-01890-s001.zip › Figure S3b.pdf]

# Breast MRI specificity in preoperative staging

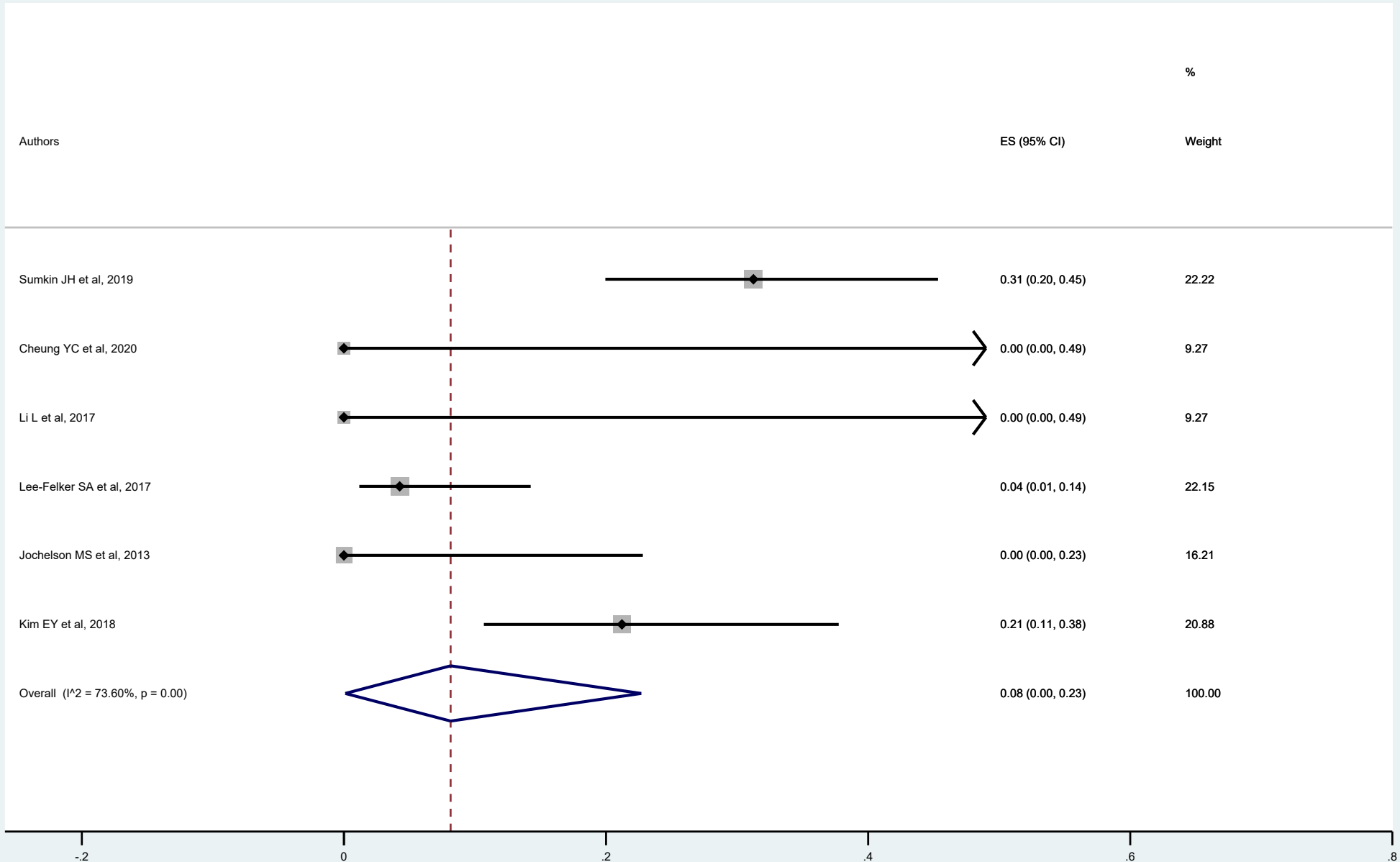

Supplement: Supplementary file 1 [file diagnostics-12-01890-s001.zip › Figure S4a.pdf]

# CEM specificity in preoperative staging

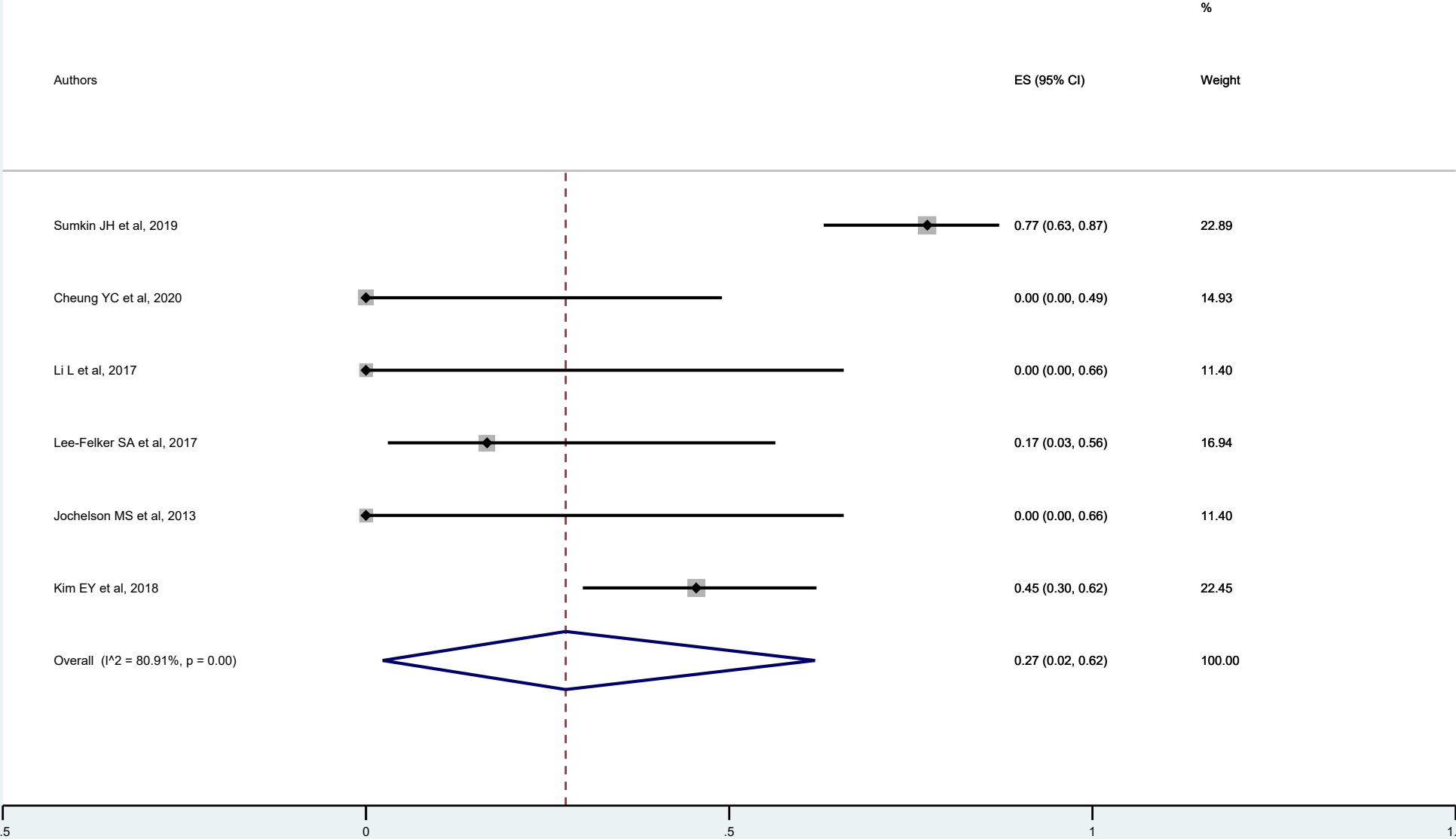

Supplement: Supplementary file 1 [file diagnostics-12-01890-s001.zip › Figure S4b.pdf]
